# Rewiring care delivery through Digital Therapeutics (DTx): a machine learning-enhanced assessment and development (M-LEAD) framework

**DOI:** 10.1186/s12913-024-10702-z

**Published:** 2024-02-23

**Authors:** Alessandro Carrera, Stefania Manetti, Emanuele Lettieri

**Affiliations:** School of Management, Politecnico di Milano, Milan, Italy

**Keywords:** Digital therapeutics, DTx, Health Technology Assessment, Framework, Machine learning, Study Design

## Abstract

**Background:**

Digital transformation has sparked profound change in the healthcare sector through the development of innovative digital technologies. Digital Therapeutics offer an innovative approach to disease management and treatment. Care delivery is increasingly patient-centered, data-driven, and based on real-time information. These technological innovations can lead to better patient outcomes and support for healthcare professionals, also considering resource scarcity. As these digital technologies continue to evolve, the healthcare field must be ready to integrate them into processes to take advantage of their benefits. This study aims to develop a framework for the development and assessment of Digital Therapeutics.

**Methods:**

The study was conducted relying on a mixed methodology. 338 studies about Digital Therapeutics resulting from a systematic literature review were analyzed using descriptive statistics through RStudio. Machine learning algorithms were applied to analyze variables and find patterns in the data. The results of these analytical analyses were summarized in a framework qualitatively tested and validated through expert opinion elicitation.

**Results:**

The research provides M-LEAD, a Machine Learning-Enhanced Assessment and Development framework that recommends best practices for developing and assessing Digital Therapeutics. The framework takes as input Digital Therapeutics characteristics, regulatory aspects, study purpose, and assessment domains. The framework produces as outputs recommendations to design the Digital Therapeutics study characteristics.

**Conclusions:**

The framework constitutes the first step toward standardized guidelines for the development and assessment of Digital Therapeutics. The results may support manufacturers and inform decision-makers of the relevant results of the Digital Therapeutics assessment.

**Supplementary Information:**

The online version contains supplementary material available at 10.1186/s12913-024-10702-z.

## Background

Chronic diseases and comorbidities affect a significant portion of the population, and older adults are particularly vulnerable [1–3]. In Italy, around 50% of people aged between 65 and 75 suffer from at least one chronic disease [4]. Italy has the highest median age among all European countries [5]. This highlights the urgent need to develop, adopt, and implement strategies that can help manage, treat, and care for older adults and their chronic health conditions [6]. This is particularly crucial considering the limited healthcare resources available and the fact that over 50% of the country’s surface area is made up of rural regions that have limited access to medical care [7, 8].

Healthcare digitalization involves using digital technologies to revolutionize the delivery, management, and accessibility of health services and information [9, 10]. Digitalization has enabled various innovations, including electronic health records, telemedicine, mobile health applications, wearable devices, artificial intelligence, and Digital Therapeutics (DTx) [9, 11–15]. These advancements have the potential to improve healthcare quality, efficiency, and equity, and achieve better outcomes, bringing benefits to patients and healthcare professionals when used effectively [16]. However, healthcare digitalization is complex and dynamic, involving various stakeholders, challenges, and opportunities [17].

This paper focuses on DTx as a specific aspect of digitalization in healthcare [18, 19]. DTx are software-based interventions that deliver evidence-based therapeutic interventions to patients using high-quality software programs to prevent, manage, or treat a medical disorder or disease. DTx have the potential to mitigate the challenges posed by chronic conditions, such as improving patient adherence, self-management, and quality of life [20, 21]. DTx for chronic diseases such as diabetes, for instance, provide patients with tailored digital coaching and insights to optimize treatment plans (e.g., BlueStar) [22, 23]. EndeaverRx, an example of DTx for the treatment of ADHD in children, is designed with a game-based approach for attention management, resulting in more engagement than other standard treatments [24]. Therefore, DTx can potentially be effective and evidence-based tools to support patients’ health [25], and it is crucial to involve patients in the development and use of these solutions to take the expected benefits [26]. The concept of value in healthcare is becoming increasingly important, with value being defined as the health outcomes that matter to patients relative to the costs of delivering such results [27–29]. This notion has driven most efforts to assess the value of health technology, including Health Technology Assessment (HTA), a process designed to promote an equitable, efficient, and high-quality health system [30]. As digital technologies continue to evolve, the healthcare sector must be ready to integrate them into processes to take advantage of their benefits, ensuring their safety and equal access to care services [31, 32].

Digital Therapeutics are digital health technologies that deliver medical interventions through software programs to prevent, manage, or treat medical disorders or diseases [20]. These products can be used alone or in combination with other therapies to improve patient care and health outcomes [21]. Although there is still a debate about the cost-effectiveness of digital technologies for health purposes, an increasing amount of ongoing evidence indicates that they have the potential to offer a cost-effective solution for managing chronic diseases. However, ongoing studies are still investigating the efficacy of these emerging technologies and no definitive evidence has been established yet [33–35]. In this sense, the development, adoption, and implementation of DTx also entail significant ethical, legal, and social challenges including data privacy and security, the digital divide and health equity, the reliability and validity of machine learning algorithms, and the need for rigorous clinical evaluation and regulation of DTx [31, 36–38]. On the dark side, it is also important to include the unintended social and behavioral impacts of DTx, such as the exacerbation of health disparities and the loss of human touch and empathy [39]. The overreliance and addiction to digital devices, as well as the ethical dilemmas and moral conflicts that may arise from the use of DTx, are additional concerns that need to be critically examined and addressed. Therefore, it is essential to ensure that the benefits of DTx outweigh the harms and that the implementation of DTx is guided by evidence-based practices and ethical principles [20, 21].

In many countries, including Italy, DTx products are considered medical devices and must be reviewed, certified (e.g., CE marking s required), and authorized by regulatory bodies to ensure their safety and effectiveness [20]. At the European level, the relevant legislation in the field of medical devices, thus including DTx, is the Medical Devices Regulation (MDR) 2017/745 [40]. According to the Regulation, a Digital Therapeutic can be considered Software as a Medical Device (SaMD), referring to the central role of the algorithm underlying their functioning [41]. Despite the growing interest in DTx, Italy has not yet produced an official definition or development pathway for these products [42]. This lack of clarity has hindered the diffusion of DTx technologies in Italy [43]. To address this issue, this study aims to provide original insights and develop a framework for developing DTx in Italy.

The paper is organized as follows: the first subsection reviews the literature on the assessment of health technologies to lay the foundation for the research framework and hypotheses. Section 2 outlines the methods of data collection and analysis. Section 3 presents the findings of the empirical investigation and discusses the significant achievements of the paper. Finally, Sect. 4 discusses the main results and their implications for researchers, managers, and policymakers.

### Assessment of health technologies

According to Tunis and Ommaya (2002), clinical research is an essential component of medical and health research to produce knowledge valuable for understanding human disease, preventing and treating illness, and promoting health [44]. Two main types of clinical studies exist: interventional (or experimental) and observational [45]. While local regulations define the characteristics of clinical research for new products (e.g., drugs, medical devices), little is known when dealing with new digital technologies such as Digital Therapeutics. With some exceptions (e.g., Germany), there is no standard or shared framework for the study design of a DTx. In the literature, it is possible to find examples of frameworks that summarize the existing study types and can help select the most suitable alternative. For instance, Grimes and Schulz (2002) developed an algorithm for classifying the kinds of clinical research that can be successfully applied to traditional medical products and technologies [46]. The framework, however, cannot be fully employed when dealing with Digital Therapeutics. Indeed, the development and assessment of disruptive innovation such as DTx introduces additional hurdles (e.g., regulatory frameworks, ethics considerations, organizational challenges) calling for the design of updated and potentially new methodologies for research and evaluation [47].

A relevant stream of literature that can be considered to frame a new health technology is the Health Technology Assessment. The main objective of HTA is to determine the actual and potential effects of given health technologies - as well as the consequences on the healthcare system, national economy, and society resulting from their adoption - both a priori and along their whole lifecycle [48]. As medical devices, pharmaceuticals, medical procedures, and health programs are the most common technologies assessed in HTA practices, Digital Therapeutics might become frequently assessed in the future [49]. Since most HTA agencies are governmental (hence, publicly funded), adopting formal HTA pathways is relevant to ensure a balance between equal and sustainable care and the adoption of innovative health technologies. Further, besides informing decision-makers, HTA has another vital role: orientating innovation in the healthcare industry and informing relevant stakeholders (e.g., clinicians, patients) of the drivers behind selecting the best health technologies. Each of the several existing frameworks (e.g., EUnetHTA Core Model, EVIDEM, and MAST) points out that to promote equitable, efficient, high-quality health, any HTA framework requires generating multidisciplinary, high-quality evidence. As Busse et al. (2002) pointed out, any HTA process should include the systematic retrieval of reliable evidence covering all the relevant domains of the assessment [50]. However, as far as digital health innovations (e.g., DTx) are concerned, traditional assessment approaches have partly failed since introducing digital health technologies disrupts the conventional paradigms of care (e.g., the patient-clinician relationship) [51, 52]. Additionally, the generalizability of results related to digital health technologies is more challenging than traditional health technologies since it is inherently context- and actors-specific [53, 54].

The present research aims to develop a framework to help fill the DTx validation gap.

## Methods

**Fig. 1:**
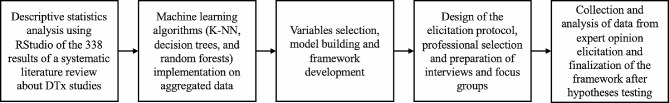
Flow chart of the methodology adopted

The study has been conducted relying on mixed methods, combining analytical and qualitative methodologies to increase the reliability of results [55] and to strengthen their evaluations [56]. Figure 1 shows the sequence of phases of the study.

### Design

**Fig. 2:**
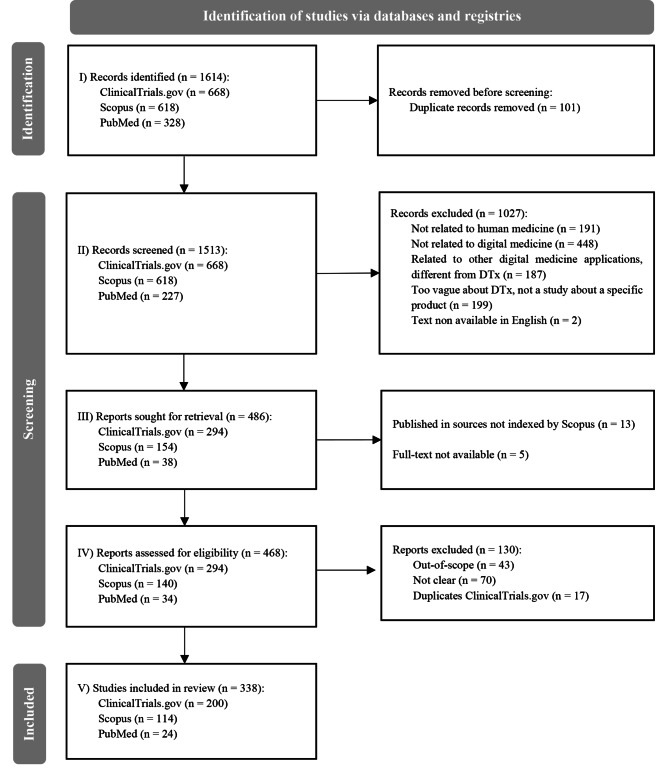
PRISMA diagram

A systematic literature review following the Preferred Reporting Items for Systematic Reviews and Meta-Analyses (PRISMA) guidelines was performed (Fig. 2) [57].

The literature search was conducted in Scopus, PubMed, and ClinicalTrials.gov databases. Search query included keywords concerning DTx (e.g., “digital therap*”, “DTx”), and only English-language records were considered. The search was constrained by documents published from 2015 onwards, considering the approval of the first American DTx (i.e., reSET) by the FDA in 2017. Records meeting the following two criteria were incorporated into the review: (1) the study aligns with the Digital Therapeutics Alliances’ [21] definition of DTx, and (2) the study explicitly reports results and implications related to DTx. Conversely, records were excluded if they met any of the following criteria: (1) lack of relevance to human medicine, (2) lack of relevance to digital medicine, (3) provision of a vague definition for DTx with a general perspective, and (4) absence of studies assessing specific DTx in their content. Two researchers (AC and SM) performed title and abstract screening and full-text reading. Disagreements have been solved through discussion between the two reviewers and by consulting a third researcher (EL).

Aggregated data from the systematic literature review were analyzed using descriptive statistics via RStudio. Then, a subset of 33 variables related to the 338 included studies was selected and divided into predictors [23] and targets [10]. Three machine learning (ML) algorithms (namely, K-NN, decision trees, and random forests) were iteratively applied to build models to predict the target variables starting from the predictors and find patterns in the data. According to the specific characteristics of the dataset, a metric for comparison of model performances, namely Model Comparison Metric (MCM), was defined based on the weighted average of accuracy (0.5), specificity (0.25), precision (0.125), and recall (0.125) [58]. As decision trees reported better MCM values than the other algorithms, their outputs, combined and interpreted considering the recommendations from the literature, were used to develop a framework for assessing Digital Therapeutics. The framework, organized in five sections, guides DTx study design and assessment.

Starting from the multiple-step procedure developed by Knol et al. (2010), the following sequence of steps was followed: (i) translation of the framework’s pillars into hypotheses in the form of statements and visual representations, (ii) definition of the scope and format of the elicitation, (iii) selection of experts, (iv) design of the elicitation protocol, (v) preparation of the elicitation session, (vi) elicitation of expert judgments, (vii) text analysis, aggregation, and reporting, (viii) feedback mechanisms to correct the framework [59]. Therefore, the paper is based on the integration of quantitative and qualitative data. The results of the literature review were analyzed through ML algorithms, allowing interpretation and attribution of meaning in the form of a framework [60]. In addition, the qualitative approach brought further insights through interviews and validated the hypothesized framework [61].

The framework was decomposed into 18 statements expressed in the form of hypotheses (Table 1).

**Table 1:** Hypotheses development and framework main components

Component	Id	Hypothesis
Study type and randomization	H1.1	Study type is affected by: (a) study needs and domain choices, (b) product development and study phase, (c) availability of sources of evidence
	H1.2	Study type is NOT affected by: (a) target disease, (b) product features.
	H2	Decisions about study randomization and control depend on: (a) study needs and domain choices, (b) product development and study phase
Patients and study duration	H3.1	Decisions about which patients to include in the studies depend on: (a) study needs and domain choices, (b) product development and study phase, (c) spread and burden of the disease
	H3.2	The number of patients to include in a study depends on: (a) study needs and domain choices, (b) product development and study phase
	H3.3	The decision about whether and how to train patients in studies depends on: (a) patients’ digital literacy, (b) patients’ awareness of the disease, (c) product ease-of-use, (d) patient’s support by healthcare ecosystem actors
	H4	The study duration depends on: (a) product development and study phases, (b) scalability of the product features, (c) target disease
Comparators and study arms	H5.1	The choice of comparators depends on: (a) target disease, (b) routine treatments for that disease, (c) patient groups, (d) product features
	H5.2	DTx based on asynchronous digital content should be compared to ‘digital placebos’ whose digital content has no therapeutic effect
	H5.3	DTx products’ active ingredients should be assessed separately, when possible, especially in early-stage studies (e.g., CBT, game, chat, alerts)
	H5.4	The effects of pharmaceuticals and medical devices to be used in addition to the DTx should be isolated
	H5.5	The number of arms/cohorts does not depend directly on the product features, but it is affected by the healthcare ecosystem actors accessing those features (e.g., patients, clinicians, caregivers, and healthcare structures)
Outcomes and scales	H6.1	Clinical evidence should be gathered through interactive product features (e.g., diary, social media, symptoms reporting) and, relying as much as possible on PROMs and validated scales
	H6.2	Quality of Life (possibly measured using non-disease-specific standard scales) should be considered in the study of any DTx. This can help in carrying out cost-utility analyses
	H6.3	Since ‘Perceived Usefulness’, ‘Usability’, and ‘Acceptability’ represent critical factors in the patient’s adoption of Digital Therapeutics, such outcomes should be included in the study of any DTx. They should be measured using both objective and subjective tests
	H6.4	Economic analyses should be carried out separately, and only after, clinical studies and analyses of the organizational impact of the DTx: what are the changes in process, structure, and culture?
	H.6.5	The patient dropout from studies and actual use of a DTx must be assessed since it might hide insights about ethical aspects (e.g., social/economic barriers) hindering the use of the digital therapy
	H6.6	«One does not fit all»: profiling target users of a digital therapy is necessary to make sure that the patient is willing to get more empowered in the management of his disease by using the DTx
Sources of evidence	H7	Study design and needed evidence must guide the strategic selection of the sources of evidence. There is a need for study plans to manage the use of RWE from a statistical standpoint

### Participants

A purposeful sampling approach has been adopted to select critical stakeholders to be involved in the expert opinion elicitation [62]. To maximize the breadth of the perspectives, it was decided to include all three types of professional experts mentioned by Knol et al. (2010) (namely generalists, subject-matter experts, and normative experts), resulting in a total of eight people with a heterogeneous background [59]. Their expertise ranged from business and product development to healthcare management, health regulatory policies, clinical research, and clinical practice. Table 2 summarizes the profiles and roles of the people involved.

**Table 2:** Professionals involved during the expert opinion elicitation

Organization	Role(s)
Italian contract research organization (CRO), leader in the development of DTx in Italy	CEO Managing Director
Italian Pharmaceutical Research Institute	Researcher specialized in Health Regulatory Policies
Italian Scientific Institute for Research, Hospitalization, and Health Care currently involved in studies about a DTx	Hospital manager Physician, research director, and full professor Psychologist and full professor
Italian MedTech company currently developing a DTx	Corporate Innovation manager
Multinational pharmaceutical corporation currently developing a DTx	Medical Evidence Advisor

In addition, contacted stakeholders have been asked to disseminate this study invitation to other profiles of interest and refer to eligible contacts (i.e., snowballing). Eligible participants needed to be experts in the field of (i) Digital Therapeutics, (ii) clinical development of new medical technologies, and (iii) involved in new digital health product development project(s).

### Data collection

Focus group discussions and semi-structured interviews have been conducted via Microsoft Teams between September and December 2022. In addition, focus group discussions and interviews were recorded and performed in Italian and were about 1–2 h long. The day before the interviews, after collecting informed consent from the participants and confirmation of their willingness to participate, an outline of the questions that would be asked during the interview was shared with them. Most were planned; however, others emerged during the sessions. Participants were allowed to answer each question and appeared engaged during the interview, sharing their experiences. The experts involved were asked to comment on the framework by highlighting its strengths and limitations and suggesting possible improvements based on their expertise. At the beginning of each interaction, the moderator (AC or SM) introduced himself and explained the purpose and procedures. Next to the moderator, the observer (SM or AC) was present in discussions and responsible for time management and taking notes. A PowerPoint presentation guided participants through the focus group discussion and interviews. Several recommendations were followed to minimize inherent biases in subjective judgment and errors related to the elicited outcomes (e.g., to avoid misperception of notions about likelihood and probability) [63].

### Data analysis

Upon completing the interview process, two researchers (AC and SM) engaged in a collaborative debriefing session to share their observations and facilitate the recording of salient details, including non-verbal behaviors. The data obtained were analyzed deductively (i.e., based on the research objectives) and inductively (i.e., based upon the recognition of patterns), relying on a process of data familiarization followed by the coding phase using Microsoft Excel, following a thematic analysis approach [64]. The verbatim transcription of each interview was accompanied by meticulous notetaking, enabling the identification of key sentences to generate codes and emergent themes. These themes were subsequently grouped, organized, and abstracted, with the attainment of data saturation and replication in themes serving as a guiding principle. Additionally, the senior researcher (EL) conducted an independent review of the transcripts to generate codes and themes. A working analytical framework (namely, a coding tree) has been created and applied to the other transcripts. Finally, the data were processed into a matrix and interpreted. The final themes, sub-themes, and relative categories were then subjected to a thorough discussion by a team of three researchers with the aim of refining and clarifying their significance by following a consensus-driven approach [65, 66].

## Results

The analytical analysis of aggregated data deriving from the systematic literature review of 338 studies about DTx has been considered for the development of M-LEAD, a Machine Learning-Enhanced Assessment and Development framework. The framework comprises five components (namely, study type and randomization, patients and study duration, comparators and study arms, outcomes and scales, and sources of evidence). The resulting framework, therefore, combines background literature, machine learning outputs, and expert opinion elicitation. An overview of the insights derived from the expert opinion elicitation is provided in Table 3.

**Table 3:** Summary of the findings from the expert opinion elicitation

Component	Hyp.	Result	Comment/remark
Study type, randomization, and control	H1.1	Supported	The correlation between the study design and the DTx development stage is in line with personal experiences of development and assessment of DTx products
	H1.2	Partly supported	It is reasonable that product features do not influence the study types, however, personal experiences suggest not to exclude that different disease clusters (e.g., chronic vs. acute) might affect the study design
	H2	Supported	The essential component of any trial before market approval should be the control, whose outcomes get more reliable in case of randomization
Patients and study duration	H3.1	Supported	Both healthy volunteers and target patients during the initial stages should be considered; later, efforts should be devoted to target patients for clinical purposes
	H3.2	Supported	The number of patients ultimately depends on the target disease characteristics (e.g., spread, incidence, presence of comorbidities), as not all the patients are suitable for the same treatment
	H3.3	Supported	The training effort is fundamental, as well as the presence of backup support provided by the familiar context or caregivers
	H4	Supported	The factors mainly affecting the study duration include the rarity of the disease and the timespan of outcome realization
Comparators and study arms	H5.1	Supported	Comparators are, by definition, dependent on product features and are often related to the target disease, as the standard of care is disease-specific
	H5.2	Supported	Using a digital placebo would be correct from a methodological standpoint, as it would allow to produce more reliable and generalizable results
	H5.3	Not supported	It would be optimal to isolate the effects of multiple mechanisms of action, but this is rarely done due to economic and time constraints. What matters, in the end, is the evaluation of the overall effect of the DTx on the patient
	H5.4	Partly supported	Even though not fundamental, an arm comparing the DTx with the standard of care could prove that digitalization improves both the efficacy and efficiency of the patient’s care pathway
	H5.5	Supported	The number of arms/cohorts is directly dependent on the patients and comparators chosen
Outcomes and scales	H6.1	Supported	Clinical evidence can be gathered using patterns analysis, relying on machine learning technology to save economic and time resources
	H6.2	Supported	There is the need to collect since the very early stages data supporting subsequent economic study phases, which might accelerate later HTA activities
	H6.3	Partly Supported	In addition, cultural change should be considered, especially by considering the patient’s willingness to pay and be responsible for their care.
	H6.4	Supported	Economic analyses depend on organizational ones and are carried out in later stages
	H6.5	Supported	Dropout and non-responders’ analyses should be included in any study protocol, and statistical plans should inform about how to address them adequately, as collecting this kind of information may help to manage product development and training initiatives better
	H6.6	Partly supported	Collecting extra data is a good practice; however, resource availability might limit this practice
Sources of evidence	H7	Supported	Study design and need of evidence guide the selection of sources of evidence. There is a need to strategically plan data collection and statistical analysis of RWE and use patterns

The following sections will focus on three of the components of the framework (namely, comparators and study arms, outcomes and scales, and sources of evidence), whose implications are the most relevant to discuss.

### Comparators and study arms: the role of digital placebos

A significant portion of the expert elicitation was dedicated to study comparators and arms. This framework section is consistent with what experts have directly experienced with the study design of products under development. Both the CRO representatives and the researcher stated that comparators depend on product features and are often related to the target disease, as the standard of care is disease-specific. Additionally, according to the CRO managing director and the medical evidence advisor, now DTx typically represent the digital edge of an existing non-digital pathway– the traditional standard of care.

“I think that an arm comparing DTx with the standard of care should exist to prove that digitalization has improved both efficacy and efficiency of the patient’s care pathway.” (1, CRO representative).

Considering the most used comparators in the literature (namely standard of care or no intervention), several participants pointed out that the standard of care and no intervention are significantly different options. No intervention might indeed be unethical and not even recommended by the regulations, as the objective of a clinical study should be to demonstrate that the product is better than - or at least equal to - the standard of care.

“Comparing a Digital Therapeutics product candidate with a no intervention arm would likely generate a positive assessment of the intervention, which may be worse than existing standards of care.” (6, psychologist).

Finally, according to the researcher, the three hospital representatives, and the CRO managing director, any DTx can have a digital placebo. Further, for the physician, digital placebos represent a potentially valuable tool for the study of DTx.

“I believe that it should not be recommended to compare completely different intervention modalities, like the DTx vs a non-digital intervention, to minimize the risk of confounding.” (5, physician).

Hence, a digital placebo would be correct from a methodological standpoint, producing more reliable and generalizable results.

### Outcomes and scales: spotlight on the organizational aspects

Another relevant part of the discussion concerned HTA domains and outcomes. When discussing this section, there was substantial agreement on the results proposed. Among the main HTA domains (namely clinical, human factor, societal, organizational, ethical, and economic), the MedTech manager presented his opinion about the organizational implications of a DTx.

“According to my experience, DTx do not determine organizational changes in terms of processes and structure, but mainly in terms of market access and regulations.” (7, MedTech company representative).

Further, cultural change, considered part of the organizational domain, is also relevant. DTx strongly affect the patient and his willingness to pay and be empowered for his care. This influence should be considered when developing business models and reimbursement policies in countries (e.g., Italy) where the propensity toward out-of-pocket healthcare expenses is limited. Hence, before economic barriers to DTx, organizational aspects, in terms of cultural change, should be carefully assessed.

### Sources of evidence: the opportunities of real-world data

As argued by both the health policy researcher and the CRO managers, study designs must support the answer to a clinical question. Hence, even in case of a lack of sources of evidence to demonstrate an outcome, it is necessary to strategically plan study design and data collection so that the evidence needed can be generated.

“It is a good practice to collect more than strictly necessary data also in early stages as such data, combined with administrative and cost information, can provide additional evidence during validation stages.” (2, CRO representative).

From this perspective, according to the CRO managers and all the hospital representatives, it is true that study design and purposes guide the selection of sources of evidence and not vice versa. Several experts stressed the importance of developing data collection, in particular of real-world data (RWD) and statistical analysis plans to correctly infer clinical, human factor, societal, organizational, economic, and ethical evidence from insightful extra data collecting during trials, which might be valuable for many purposes (included HTA) when analyzed ex-post in real-world evidence (RWE) settings.

## Discussion

The field of Digital Therapeutics (DTx) is rapidly evolving, with various stakeholders involved in the development, regulation, procurement, and use of these digital health solutions. DTx have gained significant attention in recent years for their potential to address a wide range of health conditions, from chronic illnesses to mental health disorders, and to improve the overall quality of care. However, the lack of standardized methods for assessing their multidimensional impact is a significant barrier to their adoption and integration into clinical practice and reimbursement schemes. There are various HTA frameworks available, including the EUnetHTA Core Model, EVIDEM, and MAST that is designed specifically for telemedicine. However, traditional frameworks have limitations when applied to digital health and may not fully cater to the requirements of innovative digital medicine applications, such as DTx. This gap affects not only the mainstream parameters of benefit, such as effectiveness and cost-effectiveness, but also broader parameters, like therapy adherence, user satisfaction, engagement, and organizational adaptation to real-life environments [54, 67–69].

To address this gap, our study aimed to develop and validate a comprehensive, evidence-based framework that systematically guides the development and assessment of DTx in healthcare. While we build on the existing frameworks and guidelines for Health Technology Assessment of digital health interventions, such as the one recently proposed by Tarricone et al. (2022) [68], the M-LEAD framework offers unique insights, as it is explicitly designed to address the distinct elements and criteria of DTx. The following sections provide critical insights with the final goal of establishing a common and shared framework for fostering the adoption of DTx in Italy. Each framework component has been validated, relying on interviews and focus groups. Experts’ knowledge and experience of participants improved study reliability and validity [70].

### Theoretical contributions and managerial implications

The development and validation of the M-LEAD framework is a significant contribution to the literature on Digital Therapeutics and Health Technology Assessment. The M-LEAD framework provides a comprehensive and systematic approach to guide the development and assessment of DTx, addressing the gap in the existing frameworks and guidelines for digital health interventions.

The M-LEAD framework incorporates specific elements and criteria relevant to DTx, such as selecting a suitable comparator, measuring user adherence and engagement, and assessing the ethical and organizational implications of DTx. By integrating the perspectives and preferences of the end-users and the healthcare system, the framework ensures that the DTx solutions are aligned with the needs and expectations of their users and stakeholders. What sets this framework apart is its emphasis on involving patients and other stakeholders in the co-design and co-assessment of DTx. This approach ensures that these digital interventions meet the end-user’s and healthcare system’s needs and preferences. The framework also addresses the challenges and opportunities of integrating DTx into existing care pathways and workflows. It recognizes the potential impact of DTx on the roles and responsibilities of healthcare professionals and patients.

Moreover, the framework addresses the ethical and legal issues arising from using DTx, such as data privacy and security, informed consent, liability, and accountability. Another distinctive characteristic of this framework is its focus on the potential of DTx to reduce health inequalities and improve access to care, especially for underserved and vulnerable populations. Finally, the framework underscores the need for adaptive and iterative assessment methods for DTx. This approach accounts for the dynamic and evolving nature of these interventions and their contexts of use.

The M-LEAD framework has important implications for the stakeholders involved in the DTx sector, including developers, researchers, regulators, payers, clinicians, and patients. The framework can help developers and researchers plan, design studies, and develop evidence-based products demonstrating their value and impact. It can also assist regulators and payers in developing standardized guidelines and criteria for assessing DTx, essential for governing a rapidly growing and evolving sector. The framework can also support clinicians and patients in making informed decisions about adopting and using DTx and assessing their outcomes and experiences. By promoting evidence-based and cost-effective DTx, the M-LEAD framework can potentially enhance the quality and efficiency of healthcare delivery and outcomes, benefiting patients, clinicians, payers, and society.

The M-LEAD framework also offers valuable knowledge for decision-making and policy-making processes, particularly regarding the adoption and reimbursement of DTx. It highlights the need for a coordinated and standardized approach to assessing the value and impact of DTx, considering the multidimensional and dynamic nature of these interventions and their contexts of use.

The framework also emphasizes the importance of involving and training healthcare professionals and patients to assess clinical practices associated with DTx, as they are central to the paradigm shift in the patient-clinician relationship introduced with DTx. It stresses the need to determine the users’ awareness and the ethical and organizational implications of this paradigm change.

The M-LEAD framework can help establish a common language and principles for developing and assessing DTx, enabling stakeholders to collaborate and communicate effectively. It can also foster innovation and collaboration among the stakeholders involved in the DTx sector, creating platforms and networks for sharing best practices, data, and experiences and facilitating the co-creation and co-assessment of DTx solutions. The framework can also help address the challenges and opportunities of applying the framework to different types and stages of DTx, such as prevention, diagnosis, treatment, and monitoring, and how the framework can be adapted to different contexts and settings. It can also help ensure the sustainability and scalability of the DTx solutions by addressing the gaps and uncertainties in the evidence base, incorporating the perspectives and preferences of the end-users, and ensuring the safety and quality of the DTx solutions.

### Strengths and limitations of the study and further research

This study has several strengths and limitations that need to be acknowledged. Firstly, the study has a strength in developing and validating a comprehensive and systematic framework for developing and assessing DTx, addressing the gap in the existing frameworks and guidelines for digital health interventions. The M-LEAD framework incorporates specific elements and criteria relevant to DTx, such as selecting a suitable comparator, measuring user adherence and engagement, and assessing the ethical and organizational implications of DTx. The M-LEAD framework also integrates the perspectives and preferences of the end-users and the healthcare system, ensuring that the DTx solutions are aligned with the needs and expectations of the stakeholders.

Secondly, the study has a strength in conducting the validation process in collaboration with the DTx developers, who are the primary users of the framework. The framework was refined iteratively, considering the various development phases of the DTx. Throughout the development and validation process of the framework, the technology readiness level (TRL) increased from the preliminary stages, indicating the progress and maturity of the DTx solutions. The study also involved key players in Italy, providing a comprehensive representation of the actors involved in developing DTx in the country. However, depending solely on expert opinion can lead to subjective biases that affect the consistency of the results. Nevertheless, this methodology of generating evidence is crucial given the early development stage (i.e., lower than TRL 7) of these emerging technologies [59, 71]. Future research conducted at higher TRL levels can adopt more advanced evidence-generation approaches.

Additionally, the study also has other limitations that need to be addressed. Firstly, the experts consulted to conduct this research were only from Italy, so the findings may not apply to other regions and countries. The DTx sector is rapidly evolving and diverse, with different regulatory and reimbursement frameworks, market dynamics, and stakeholder preferences. Even though the expert selection process was systematic in mapping all the DTx under development close to the Italian market, there is a potential limitation in fully capturing the diversity of opinions and experiences in the target field, with the risk of incurring representation bias. Therefore, further research is needed to update and validate this framework in different contexts and settings and to test its applicability and feasibility in real-world scenarios.

Secondly, the developed framework was based on the current state of the art of DTx and HTA, which may change over time as new technologies and methodologies emerge. The M-LEAD framework is intended to be a flexible and adaptable tool that can accommodate the dynamic and evolving nature of DTx and their contexts of use. However, the framework may need to be revised and updated periodically to reflect the latest evidence and best practices in the field. Therefore, further research is required to monitor and evaluate the framework’s performance and impact and identify the areas for improvement and innovation.

## Conclusions

The development and assessment of Digital Therapeutics (DTx) is an emerging field that has gained significant attention in recent years. DTx products are software-based interventions that aim to prevent, manage, or treat various health conditions and diseases. However, despite the growing interest in DTx, there is a significant gap in the literature regarding the development and assessment of these products. To address this gap, this study has created a new framework that seizes an opportunity and contributes to filling the relevant literature gap about DTx product development and assessment. The proposed framework, called M-LEAD, has been improved and validated using expert elicitation and represents the first step towards creating standardized guidelines for developing Health Technology Assessment of Digital Therapeutics.

The M-LEAD framework consists of principles and guidelines that can be used to assess the effectiveness, safety, and usability of DTx products. These guidelines are intended to be flexible enough to accommodate the unique features of different DTx products while still providing a standardized framework for their assessment. The M-LEAD framework has been validated through expert elicitation, which involved a group of experts in the field of DTx product development and assessment. The experts reviewed the proposed framework and provided feedback on its clarity, completeness, and usefulness. The feedback was used to refine the framework and ensure that it accurately reflects the current state of the field.

In conclusion, the proposed M-LEAD framework for DTx product development and assessment is an essential contribution to the field. It provides a standardized approach to assessing the multidimensional impact of DTx products in real-world environments, which will help to ensure that these products are developed and assessed consistently and transparently. The M-LEAD framework also represents the first step towards creating standardized guidelines for developing health technology assessments of Digital Therapeutics. Some of the original insights that the M-LEAD framework offers are: (i) incorporation of the perspectives and preferences of the end-users and the healthcare system, ensuring that the DTx solutions are aligned with the needs and expectations of the stakeholders; (ii) emphasis on the measure of user adherence and engagement, as they are critical factors for the success and sustainability of DTx products; (iii) inclusion of the ethical and organizational implications of DTx, such as data privacy and security, informed consent, liability, and accountability, and guidance on how to address them; (iv) co-design and co-assessment of DTx products, involving patients and other stakeholders in the development and assessment process, to ensure that the DTx products are user-centered and evidence-based.

### Electronic supplementary material

Below is the link to the electronic supplementary material.

Supplementary Material 1

## Data Availability

The datasets generated and analyzed during the current study are not publicly available. Although identifying information has been removed, authors cannot risk identification by making the data available for public inspection, as anonymity to respondents was guaranteed. Datasets could be available from the corresponding author on reasonable request.

## Abbreviations

CRO: Contract Research Organization
DTx: Digital Therapeutics
HTA: Health Technology Assessment
K-NN: K-Nearest Neighbors
ML: Machine Learning
MCM: Model Comparison Metric
MDR: Medical Devices Regulation
RWD: Real-World Data
RWE: Real-World Evidence
SaMD: Software as a Medical Device
TRL: Technology Readiness Level
