# Supplementary material for: Rewiring care delivery through Digital Therapeutics (DTx): a machine learning-enhanced assessment and development (M-LEAD) framework
Source: BMC Health Serv Res. 2024 Feb 23;24:237. doi: 10.1186/s12913-024-10702-z (PMC10885456; doi:10.1186/s12913-024-10702-z)
Supplement: Supplementary file 1 — Supplementary Material 1 [file 12913_2024_10702_MOESM1_ESM.docx]

**Supplementary material**

The semi-structured interviews were conducted to allow interviewees to share their knowledge and opinions with a high degree of freedom. The authors tried not to bias the responses and influence participants’ responses. Therefore, the researchers asked for interviewees’ views about the hypotheses made in this study.

- What are the main characteristics that mainly affect the study type for Digital Therapeutics?
- What are the characteristics that affect the least study type for Digital Therapeutics?
- What drives the decisions about study randomization and control for Digital Therapeutics?
- What decisions about which patients to include in the studies depend on?
- What does the number of patients to include in a study for Digital Therapeutics depend on?
- What does the decision about whether and how to train patients in a study for Digital Therapeutics depend on?
- What does study duration depend on?
- What does the choice of comparators depend on?
- What should DTx based on asynchronous digital content be compared to?
- How should the active ingredients of DTx products be assessed in a study for Digital Therapeutics?
- How do the effects of pharmaceuticals and medical devices be used in addition to the DTx should be considered in a study for Digital Therapeutics?
- What does the number of arms/cohorts depend on?
- How should clinical evidence be gathered in a study for Digital Therapeutics?
- What about quality of Life in a study for Digital Therapeutics? How could it be measured?
- Should ‘Perceived Usefulness’, ‘Usability’, and ‘Acceptability’ be included in a study for Digital Therapeutics? How could they be measured?
- How should economic analyses be carried out in a study for Digital Therapeutics? What is the relationship between clinical studies and analyses of the organizational impact of the DTx?
- Should the patient dropout from studies and actual use of a DTx be assessed?
- How do you profile target users of Digital Therapeutics in a clinical study setting?
- How are sources of evidence selected?
